# Repurposed drugs as histone deacetylase 8 inhibitors: Implications in cancer and neuropathological conditions

**DOI:** 10.3389/fphar.2024.1488585

**Published:** 2024-11-14

**Authors:** Mohammed Alrouji, Kumar Venkatesan, Mohammed S. Alshammari, Fahad A. Alhumaydhi, Sheeba Shafi, Sharaf E. Sharaf, Moyad Shahwan, Anas Shamsi

**Affiliations:** ^1^ Department of Medical Laboratories, College of Applied Medical Sciences, Shaqra University, Shaqra, Saudi Arabia; ^2^ Department of Pharmaceutical Chemistry, College of Pharmacy, King Khalid University, Abha, Saudi Arabia; ^3^ Department of Clinical Laboratory Sciences, College of Applied Medical Sciences, Shaqra University, Shaqra, Saudi Arabia; ^4^ Department of Medical Laboratories, College of Applied Medical Sciences, Qassim University, Buraydah, Saudi Arabia; ^5^ Department of Nursing, College of Applied Medical Sciences, King Faisal University, Al Hofuf, Saudi Arabia; ^6^ Pharmaceutical Sciences Department, College of Pharmacy, Umm Al-Qura University, Makkah, Saudi Arabia; ^7^ Centre of Medical and Bio-allied Health Sciences Research, Ajman University, Ajman, United Arab Emirates

**Keywords:** neuropathological conditions, drug repurposing, small-molecule inhibitors, virtual screening, cancer

## Abstract

Histone deacetylase 8 (HDAC8) is a member of class I histone deacetylases (HDACs) that catalyzes the deacetylation of both histone and non-histone proteins. Dysregulation and overexpression of HDAC8 are implicated in the development of various complex diseases, including cancer and neurodegenerative disorders. HDAC8 plays a significant role in cancer progression, contributing to cancer cell proliferation, metastasis, immune evasion, and drug resistance. The available HDAC8-targeting inhibitors suffer from poor target engagement and low tolerability, and demonstrate off-target toxicity due to limited selectivity, leading to adverse effects in patients, and thus urging for the identification and development of new molecules. Drug repurposing is a useful strategy for identifying useful drugs for predefined targets which can be exploited here for identifying promising drug molecules against HDAC8. This study involved an integrated virtual screening against HDAC8 using the DrugBank database to identify repurposed drugs capable of inhibiting HDAC8 activity. The process started by selecting the top 10 drug molecules based on their binding affinity. The drug profiling and biological function of selected molecules were then evaluated, showing anti-cancer and anti-neurological properties with a high probability of being active. Interaction analysis revealed crucial binding of radotinib and sertindole molecules with the HDAC8 protein. Both molecules showed higher binding affinity than reference inhibitor droxinostat. The elucidated molecules were further evaluated for 500 ns long-run molecular dynamics (MD) simulation with HDAC8. Structural deviation, compactness, folding behavior, hydrogen bonds analysis, and secondary structure content profiling revealed complex stability formed by HDAC8 and the selected compounds. Principal component analysis and Gibbs free energy calculations strongly recommend that both complexes were highly stable during the simulation. Overall, the results indicate that radotinib and sertindole can be promising candidates as HDAC8-targeting repurposed drugs against cancer and neuropathological conditions.

## Introduction

Histone deacetylases (HDACs) also referred to as lysine deacetylases (KDACs) are proteolytic enzymes that depend on either zinc (Zn^2+^) or nicotinamide adenine dinucleotide (NAD^+^) (Ruijter et al., 2003). These enzymes are involved in the process of transcriptional repression and chromatin condensation by stripping off the acetyl groups from the ε-amino group of lysine residues on histones and other proteins (Bannister and Kouzarides, 2011; Van Dyke, 2014). HDACs participate in various cellular processes, including cell proliferation, cell death, neuronal differentiation, and DNA replication (Reichert et al., 2012). Moreover, they have been associated with the development and worsening of several diseases and pathological states such as neurological diseases, fibrosis, cancer, metabolic disturbances, and parasitic diseases (Wiech et al., 2009; Falkenberg and Johnstone, 2014). Among all the HDAC enzymes, histone deacetylase 8 (HDAC8) is a class I HDAC that has attracted much interest because of its roles in various physiological and pathological processes (Kim et al., 2022). HDAC8 plays a role in modulating chromatin structure and gene expression on cell cycle regulation, differentiation, and survival (Chen et al., 2011).

Dysregulation of HDAC8 has been implicated in the pathogenesis of several diseases, most notably cancer and neurodegenerative disorders (Chakrabarti et al., 2016). HDAC8 is especially involved in several features of cancer development such as cell division, spreading, immune system avoidance, and chemotherapeutic drug resistance. In cancer, HDAC8 plays a role in cancer development through cell proliferation, metastasis, immune tolerance, and chemoresistance (Kim et al., 2022). Elevated levels of HDAC8 have been reported in many cancers such as breast cancer, neuroblastoma, and acute myeloid leukemia, and these have been associated with shorter survival and more aggressive disease (Chakrabarti et al., 2016). The role played by HDAC8 in neurodegenerative diseases, although not studied to a vast extent, is also significant. HDAC8 removes the acetyl group from specific proteins in neural cells that may result in neurotoxicity and the development of diseases such as Alzheimer’s disease and Parkinson’s disease (Geng et al., 2023; Li et al., 2022).

Although HDAC8 has been characterized to play an essential role in these diseases, selective HDAC8 inhibitors have been challenging to develop (Fontana et al., 2022). Recently, the use of HDAC inhibitors (HDACis) has been contemplated in many neurological disorders apart from malignant and X-linked disorders (Pal et al., 2023). The dysregulation of histone acetylation homeostasis leads to the development of psychiatric disorders, neurodegenerative diseases, and other comorbid neurological disorders (Meng et al., 2023). The current HDAC8 inhibitors have various limitations as they cause off-target effects which reduce their clinical effectiveness and have side effects (Rajaraman et al., 2023). This has brought the need for the discovery of new, selective HDAC8 inhibitors with better pharmacokinetic properties.

One potential strategy to overcome this problem is the concept of drug repurposing, which implies the search for new applications for existing drugs (Parvathaneni et al., 2019). This approach can save a lot of time in drug development and is also cheaper as the safety of the drugs being used is already known. Drug repurposing has also been useful in finding new treatments for several diseases such as cancer and neurological disorders (Pushpakom et al., 2019). Another approach to drug repurposing involves screening compound libraries to get active drugs with therapeutic potential. Virtual screening has also been identified to be a very efficient process in the drug discovery process which is used to identify new hit compounds from large numbers of compounds using computational methods (Mohammad et al., 2020). Molecular docking is one of the most practiced virtual screening methods that predict the binding affinity of a ligand to a protein (Shamsi et al., 2019). The objective of this study was to identify compounds that could act as inhibitors against HDAC8 through drug site targeting from the libraries of FDA-approved drugs. The goal of drug repositioning is to pinpoint molecules that can modulate HDAC8 and associated diseases without adverse effects.

In the present work, the HDAC8 protein was considered for structure-based drug repurposing. The screening process used in this study was an integrated approach where the first step was molecular docking. It assisted us in the determination of the appropriate drug molecules to interact with the HDAC8 protein. Such studies are useful in drug discovery and repositioning because they are cheaper and faster than the conventional approaches. Therefore, we obtained a set of 3,500 FDA-approved drug molecules from the DrugBank database (Knox et al., 2024). The best drug molecules were chosen according to the binding affinity and the extent of interaction with HDAC8. The screened molecules were further analyzed for their drug profiles and biological activity prediction. In addition, a comparison of their docked complexes with HDAC8 was performed at the atomic level by molecular dynamics (MD) simulations and further by essential dynamics. This approach helped us to select the most promising compounds for further study and possible usage as HDAC8 inhibitors.

## Materials and methods

### Molecular docking screening

Virtual screening employing molecular docking was used to select molecules that have a high binding affinity to HDAC8. For molecular docking studies, AutoDock Tools (Huey et al., 2012) and InstaDock (Mohammad et al., 2021) were used as they are very reliable in docking screening as confirmed in previous studies. Some other tools used for docking and analyzing output files were PyMOL (DeLano, 2002) and Discovery Studio Visualizer (Visualizer, 2005). The crystal structure of HDAC8 in three-dimensional conformations was downloaded from the Protein Data Bank (accession number: 5VI6) and was prepared for the docking studies using InstaDock and AutoDock Tools. Some of the preprocessing steps included fixing missing residues, the addition of hydrogens to the polar atoms, and then assigning the correct atom type to match the structure for the process of molecular docking. A set of drugs in the three-dimensional form was collected from the DrugBank database and then sorted and prepared in InstaDock v1.2. Simulations of docking were performed in InstaDock with the grid size of 71, 73, and 70 Å with the center at coordinates 5.739, −5.541, and 15.845 for the X-, Y-, and *Z*-axes, respectively. After the docking study, the log files and out files were generated for all compounds where they were ranked based on their binding affinity toward HDAC8.

### Biological potential and interaction analysis

The prediction of activity spectra for substances (PASS) analysis was used for the SAR analysis of the screened compounds for the prediction of pharmacological effects. PASS prediction offers a brief idea about probable biological activities for a compound, which is measured in terms of “the probability to be active (Pa)” and “probability to be inactive (Pi)” (Filimonov et al., 2014). If the Pa value is high, it means that the molecule is likely to possess the biological property as predicted. After the PASS analysis, the interaction mechanism and binding prototypes of the screened molecules were studied. Using PyMOL, polar contacts between the selected molecules and HDAC8 were identified. Discovery Studio Visualizer was also used for further analysis of the possible interactions of the screened compounds within the binding pocket of HDAC8. The molecules that had interactions with critical residues were selected for further studies.

### Molecular dynamics simulation protocol

MD simulation is a useful tool for the prediction of ligand–target interactions when the flexibility of the target is taken into consideration with time (Naqvi et al., 2018). It entails releasing the atoms and molecules of the whole complex and letting them move and interact in a certain manner within a certain area for a predefined. The force of interacting atoms is computed through molecular mechanics with defined force fields for the potential energy. Here, the MD simulations of the protein and protein–ligand complex with the lowest binding energy pose were performed using the GROMACS 2022.4 version (Van Der Spoel et al., 2005). Topology files were generated using the CGenFF web server, the force field applied was charmm36-jul2022 (Huang and MacKerell, 2013), and the water model used was TIP3P (Mark and Nilsson, 2001). Eight NA^+^ ions were added to make the system charge-neutral. Then, the steepest descent and simulated annealing minimizations were performed to remove any possible overlaps (Jaidhan et al., 2014). Then, the equilibration in NVT and NPT ensembles for 1,000 ps was performed. The final run of the production for the time of 500 ns was at the temperature of 298K. The obtained trajectory files were analyzed with the help of GROMACS inbuilt tools, and several parameters like energy, deviation, fluctuation, and compactness were calculated and plotted in XMGrace (Turner, 2005).

### Principal component and free energy landscape analyses

The conformational motions in a protein molecule can be described by the principal components of the trajectory obtained in the MD simulation (Papaleo et al., 2009). This is performed by clustering the motions of atoms and generating a covariance matrix that is then diagonalized to get eigenvectors and eigenvalues that represent the energetic contribution of certain components. The gmx covar command was used to calculate the covariance matrix of the *C*α atomic coordinates of protein HDAC8 before and after its binding with the selected compounds. This matrix was diagonalized to find the eigenvectors and eigenvalues of the matrix. The first two principal components, namely, PC1 and PC2, were generated with the help of gmx anaeig command. At the same time, Gibbs free energy landscapes (FELs) were used in determining the thermodynamics and folding mechanism of the protein–ligand complexes. The Gibbs free energy was computed using the gmx sham module of the GROMACS suite.

## Results and discussion

### Molecular docking screening

Molecular docking is one of the most used techniques in the drug discovery process due to its ability to predict the binding mode of the protein–ligand complex (Naqvi et al., 2018). In this study, a library of 3500 FDA-approved drug molecules was obtained from the DrugBank repository. The docking screening was carried out using the InstaDock tool to select molecules that have good binding affinity for HDAC8. Subsequently, the top 10 hit molecules that showed the best docking scores against HDAC8 were chosen (Table 1). These selected molecules exhibited appreciable binding affinity to HDAC8. The docking scores for the 10 best hits varied from −8.6 to −9.2 kcal/mol. The docking score is used to estimate how well a specific ligand interacts with the protein target, and the lower the score, the better the binding is. All the selected molecules have better binding affinity than reference inhibitor droxinostat (Liu et al., 2016) toward HDAC8, which has an affinity of −6.0 kcal/mol. The outcomes highlighted the possibility of all the identified hits as potential competitors to HDAC8, which makes these compounds potential candidates for further research and development of new HDAC8 inhibitors.

**TABLE 1:** List of screened hits against HDAC8 and their docking parameters.

S. No.	Drug molecule	Binding affinity (kcal/mol)	pKi	Ligand efficiency (kcal/mol/non-H atom)	Torsional energy
1	Bisdequalinium chloride	−9.2	6.75	0.2091	0
2	Alectinib	−9.1	6.67	0.2528	0.9339
3	Sertindole	−9.0	6.6	0.2903	1.5565
4	Dutasteride	−8.9	6.53	0.2405	1.2452
5	Radotinib	−8.9	6.53	0.2282	2.1791
6	Pimozide	−8.8	6.45	0.2588	2.1791
7	Lumacaftor	−8.7	6.38	0.2636	1.8678
8	Ponatinib	−8.7	6.38	0.2231	2.1791
9	Perflunafene	−8.6	6.31	0.3071	0
10	Bagrosin	−8.6	6.31	0.3909	0.3113
11	Droxinostat	−6.0	4.4	0.375	1.8678

### PASS analysis

The PASS server for predicting the biological activity of a given molecule is based on the prediction of activity spectra for substances (Filimonov et al., 2014). In this study, PASS analysis was used to predict the biological activity of the molecules that were selected from the docking screening. Out of the 10 molecules tested, four, namely, alectinib, sertindole, radotinib, and ponatinib, stood out as positive hits in the drug profiling. Analyzing the PASS results, it was found that two compounds, namely, radotinib and sertindole, possess high potential in anti-cancer and anti-neurodegenerative disease therapy (Table 2). Notably, the probability of a molecule possessing the expected biological property is considered high when the Pa is greater than the Pi value. Radotinib and sertindole showed relatively high values of Pa for treating neurological disorders, which ranged from 0.438 to 0.802. The PASS analysis results pointed out that radotinib and sertindole are molecules with desirable biological profiles. These molecules can be further examined for their specific interactions in drug repurposing for targeting HDAC8.

**TABLE 2:** PASS analysis of the selected molecules with their predicted activity.

S. No.	Drug	Pa	Pi	Activity
1	Alectinib	0.533	0.049	Neurotransmitter uptake inhibitor
		0.416	0.052	Heat shock protein 27 antagonist
		0.474	0.151	Anti-eczematic
		0.373	0.073	Chemosensitizer
		0.327	0.073	MAP3K5 inhibitor
2	Sertindole	0.802	0.017	Anti-neurotic
		0.721	0.007	Anti-depressant
		0.717	0.006	Mood disorders treatment
		0.693	0.007	Anti-psychotic
		0.438	0.051	Neurodegenerative diseases treatment
3	Radotinib	0.790	0.005	Protein kinase inhibitor
		0.745	0.029	Nootropic
		0.624	0.009	Angiogenesis inhibitor
		0.529	0.010	Alzheimer’s disease treatment
		0.460	0.043	Neurodegenerative diseases treatment
4	Ponatinib	0.560	0.014	Angiogenesis inhibitor
		0.454	0.039	Autoimmune disorders treatment
		0.476	0.079	Anti-neoplastic
		0.417	0.021	MAP3K5 inhibitor
		0.439	0.052	PDGFR kinase inhibitor
5	Droxinostat	0.467	0.005	Anti-neoplastic (sarcoma)
		0.548	0.088	Membrane integrity agonist
		0.472	0.031	AR expression inhibitor
		0.257	0.004	Histone deacetylase inhibitor
		0.337	0.105	Apoptosis agonist

Pa, probability to be active; Pi, probability to be inactive.

### Interaction analysis

**FIGURE 1:**
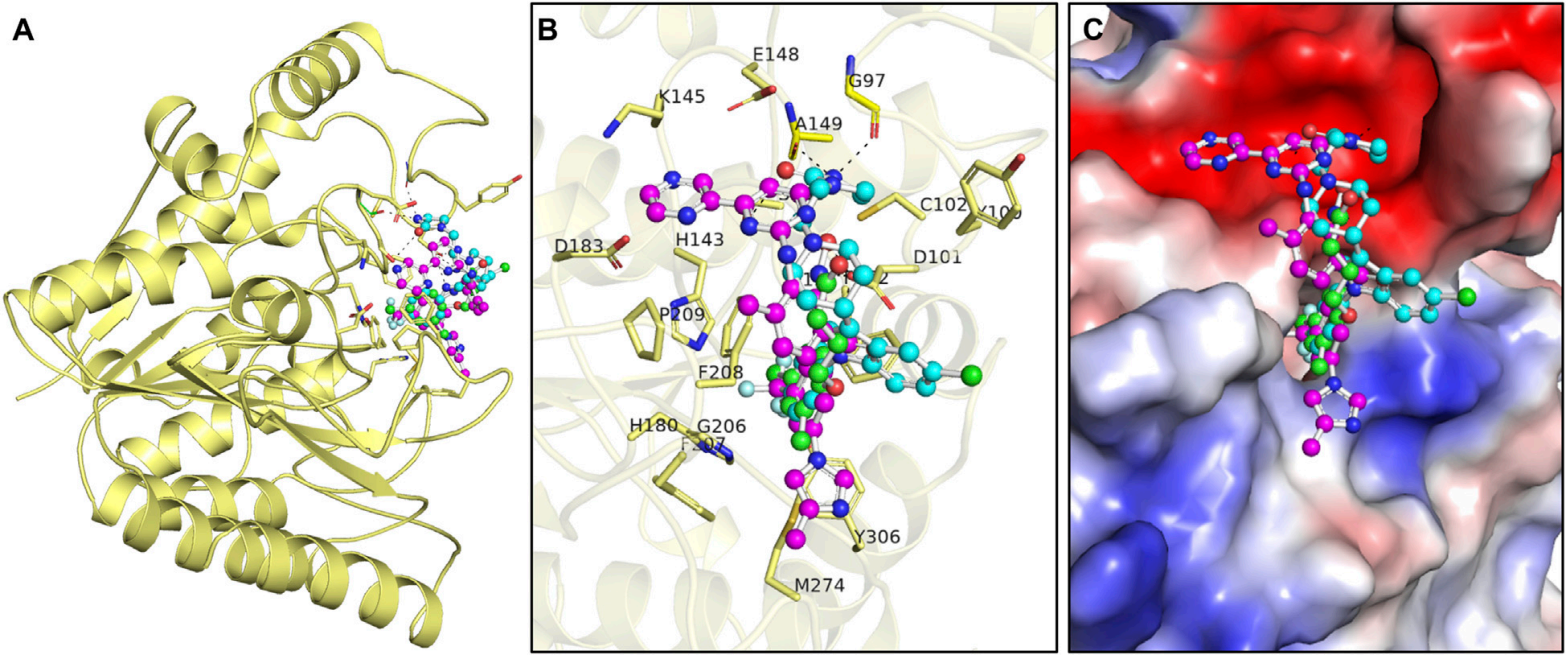
Protein–ligand interaction pattern. **(A)** Interaction pattern of HDAC8 and its interaction with droxinostat (green), radotinib (magenta), and sertindole (yellow). **(B)** Magnified view of HDAC8 binding pocket occupied by docked droxinostat, radotinib, and sertindole. **(C)** Surface potential view of HDAC8 binding pocket occupied by docked droxinostat, radotinib, and sertindole.

When repurposing drugs for new targets, it is essential to analyze the interactions within protein–ligand complexes to ensure desired efficacy and minimize off-target effects (Shamsi et al., 2024a). The drug molecules selected by assessing binding affinity and biological functions in PASS analysis were subjected to find interacting amino acid residues of the HDAC8 protein (Figure 1). PyMOL and Discovery Studio Visualizer were utilized to visualize interactions between HDAC8–droxinostat, HDAC8–radotinib, and HDAC8–sertindole complexes. The docking simulations generated 27 conformers of the selected drugs, radotinib and sertindole, as well as reference inhibitor droxinostat, bound to HDAC8, providing detailed insights into their interaction patterns (Figure 1A). Radotinib and sertindole exhibited several key interactions and the most favorable binding modes within the HDAC8 binding pocket, similar to droxinostat (Figure 1B). Both compounds occupied the active site of HDAC8 and were superimposed onto droxinostat (Figure 1C) (Dowling et al., 2008). These findings indicate that radotinib and sertindole have significant potential as HDAC8 inhibitors, warranting further drug development.

**FIGURE 2:**
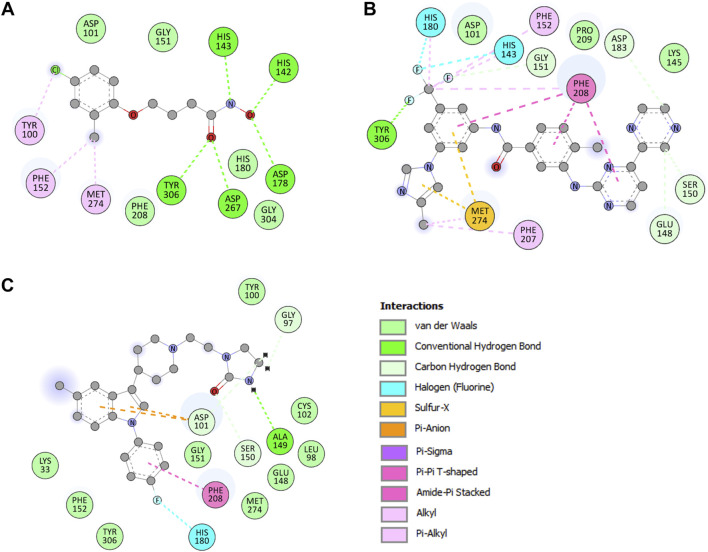
Binding residues of HDAC8 and their interactions with **(A)** droxinostat, **(B)** radotinib, and **(C)** sertindole.

Detailed interaction of reference co-crystallized molecule droxinostat showed that it formed three types of bonds, such as hydrogen bonds by His142, His143, Asp178, Asp267, and Tyr306 residues; alkyl and Pi-alkyl with Tyr100, Phe152, and Met274; and Van der Waals interactions with Asp101, Gly151, His180, Phe208, and Gly304 residues (Figure 2A). The results showed that radotinib formed many interactions with HDAC8, such as hydrogen bonds with Glu148, Ser150, Gly151, Asp183, and Tyr306 residues; halogen (fluorine) with His143 and His180 residues; and sulfur-X bond with Met274 residue (Figure 2B). It also formed Pi–Pi T-shaped bond with Phe208; alkyl and Pi-alkyl with His143, Phe152, Phe207, Phe208, and Phe208 residues; and Van der Waals interactions with Asp101, Lys145, and Gly151 residues. Similarly, sertindole made several interactions with HDAC8, including hydrogen bonds with Gly97, Asp101, Ala149, and Ser150 residues; and halogen (fluorine) by His180 residue (Figure 2C). It also formed Pi-anion formed by Asp101 residue; Pi–Pi T-shaped bond with Phe208; and Van der Waals with Lys33, Leu98, Tyr100, Cys102, Glu148, Gly151, Phe152, Met274, and Tyr306 residues. The plots showed that radotinib forms a direct close interaction with active site residue His143 of HDAC8 (Dowling et al., 2008). It also forms a hydrogen bond with the substrate binding sites Asp101 and Gly151 (Dowling et al., 2008). Both radotinib and sertindole molecules share several common interacting residues with HDAC8. These findings indicate that radotinib and sertindole be further investigated for their binding potential in MD simulation studies. Similarly, sertindole forms a direct hydrogen bond interaction with the substrate binding site Asp101 and a halogen interaction with a divalent metal cation binding site His180 (Vannini et al., 2007). Overall, the interaction analysis showed that radotinib and sertindole have a high potential to inhibit HDAC8, which can be further explored in further dynamic simulation analysis.

### MD simulation analysis

MD simulations have been widely used in molecular biology and drug discovery, and their importance has increased over the years (Shamsi et al., 2024b). MD simulations give the time evolution of atomic displacements in a protein or any other molecular system and are based on a detailed model of the physical laws that govern interatomic forces (Vlachakis et al., 2014). These simulations can reproduce a broad spectrum of the essential biomolecular transformations, such as conformational transitions, ligand binding, and folding, with the femtosecond time resolution and the description of the positions of all the atoms participating in these processes. In the present work, we have carried out a 500-ns MD simulation of four systems employing the charmm36-jul2022 force field and the tip3p water model. Using the trjconv module, GROMACS trajectories were calculated for the HDAC8 protein and protein–ligand complexes. At first, the kinetic energy of the HDAC8, HDAC8–droxinostat, HDAC8–radotinib, and HDAC8–sertindole systems was calculated and found to be 141,068 kJ/mol, 101,676 kJ/mol, 101,608 kJ/mol, and 101,679 kJ/mol, respectively. The calculated values of the potential energy of the HDAC8, HDAC8–droxinostat, HDAC8–radotinib, and HDAC8–sertindole systems were equal to −705,872 kJ/mol, −488,301 kJ/mol, −487,860 kJ/mol, and −488,180 kJ/mol respectively. The total energy of the systems formed by HDAC8, HDAC8–droxinostat, HDAC8–radotinib, and HDAC8–sertindole systems were −564,804 kJ/mol, −386,626 kJ/mol, −386,252 kJ/mol, and −386,501 kJ/mol, respectively. The three energies of the complexes were noted to be lesser than those of the HDAC8 protein, which ensured the stability of the complexes. Furthermore, the time-evolution dynamic of various parameters was calculated and evaluated as discussed in the ensuing sections (Table 3).

**TABLE 3:** Average values for various parameters computed after 500 ns simulation trajectory analysis.

System	RMSD (nm)	RMSF (nm)	Rg (nm)	SASA (nm2)	Intramolecular H-bonds
HDAC8	0.25	0.10	2.03	164.8	252
HDAC8–droxinostat	0.27	0.10	2.04	167.8	247
HDAC8–radotinib	0.23	0.11	2.01	164.2	251
HDAC8–sertindole	0.32	0.12	2.05	171.8	241

### Structural stability profile

**FIGURE 3:**
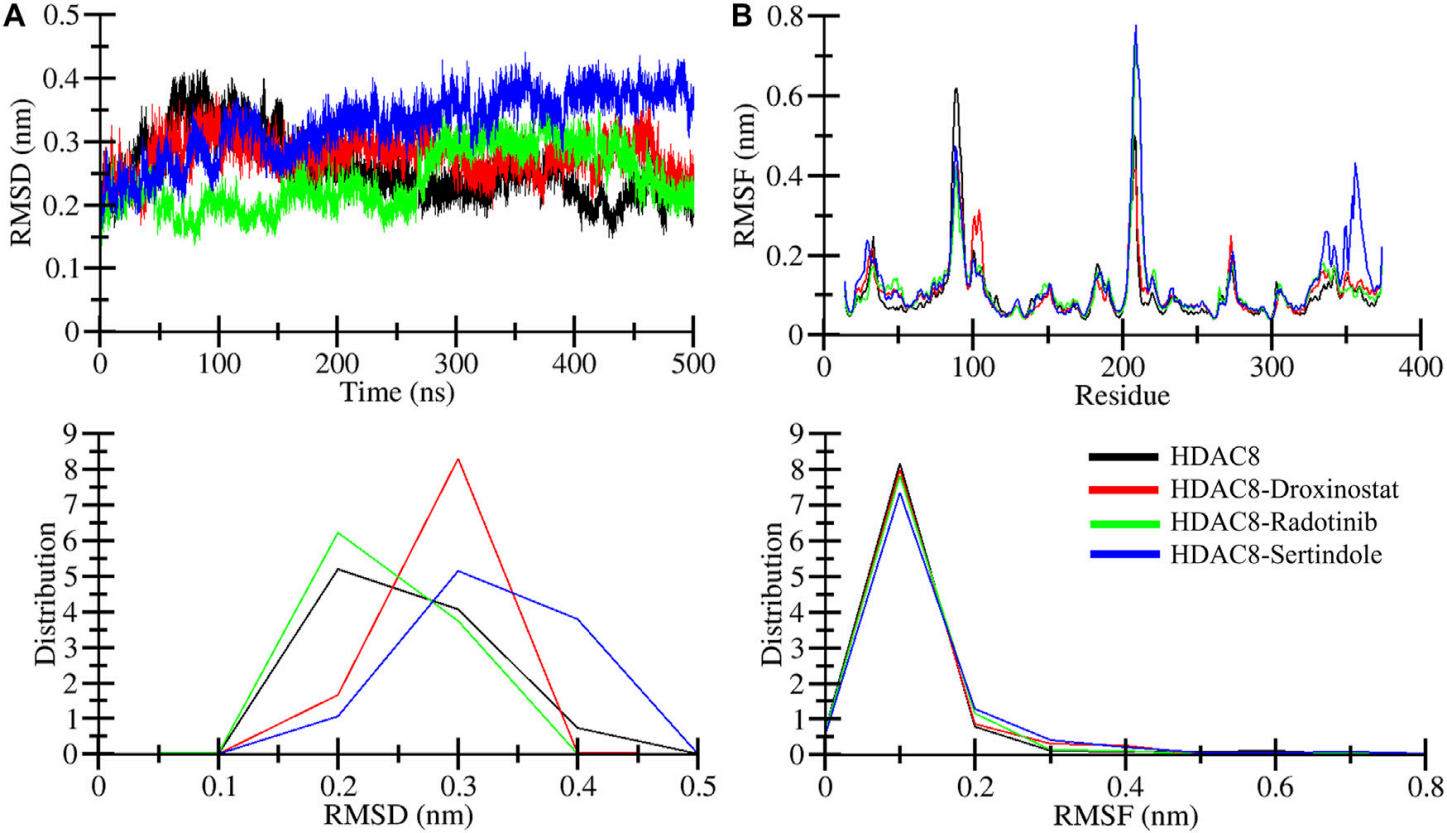
Structural dynamics of free and ligand-bound HDAC8 during the 500 ns molecular dynamics simulation. **(A)** Root mean square deviation (RMSD) plotted as a function of time and **(B)** per-residue average backbone RMSF profiles derived from MD trajectories. The lower panels show the distributed RMSD and RMSF profiles.

Root mean square deviation known as RMSD is a key phenomenon in recording structural deviation in proteins during simulation (Maiorov and Crippen, 1994). The average RMSD value was calculated for all systems to assess the average structural deviation during simulation. The HDAC8, HDAC8–droxinostat, HDAC8–radotinib, and HDAC8–sertindole systems possess 0.25 nm, 0.27 nm, 0.23 nm, and 0.32 nm, respectively (Table 3). Reference complex HDAC8–droxinostat and HDAC8–sertindole got a bit higher mean value, whereas the HDAC8–radotinib complex value recorded less than the HDAC8 protein. The maximum RMSD value also calculated for HDAC8, HDAC8–droxinostat, HDAC8–radotinib, and HDAC8–sertindole systems was 0.41 nm, 0.37 nm, 0.36 nm, and 0.44 nm, respectively. The RMSD plot in Figure 3A showing the HDAC8–sertindole complex took a small drift after 250 ns, resulting in increased deviation. The HDAC8–radotinib complex plot in green exhibits decreases RMSD from starting to around 265 ns, for a shorter time from 285 ns to 365 ns. The HDAC8–sertindole complex plot was observed a bit higher than the free protein and reference complex after 200 ns, which might be due to binding adjustment. In Figure 3A, lower panel, the distribution plot also indicates varying deviation points of the systems. The overall result indicates no major deviation occurred in both complexes which suggested the stability of the systems.

Root means square fluctuation known as RMSF is another widely performed analysis to measure residual fluctuations in the protein structure during simulation time. Individual residue fluctuations were computed for HDAC8–radotinib and HDAC8–sertindole complexes in reference to the HDAC8 protein and the HDAC8–droxinostat complex presented in Figure 3B by different colors. Around residues 85–95 higher fluctuation was measured of the HDAC8 protein than complexes shown by black. Between 203 and 214 residues of HDAC8–radotinib and HDAC8–sertindole complexes were observed higher than the HDAC8 protein and the HDAC8–droxinostat complex. The residues between 350 and 364 were seen slightly higher than all three systems. Furthermore, the average RMSF values of HDAC8, HDAC8–droxinostat, HDAC8–radotinib, and HDAC8–sertindole systems were calculated as 0.10 nm, 0.10 nm, 0.11 nm, and 0.12 nm, respectively (Table 3). In addition, the distribution of RMSF also exhibits minor variations in the fluctuations of all three systems. The observed result evaluates that HDAC8–radotinib and HDAC8–sertindole complexes got stabilized without any major drift, and showed a similar pattern of fluctuations as the free protein.

### Assessment of structural folding behavior

**FIGURE 4:**
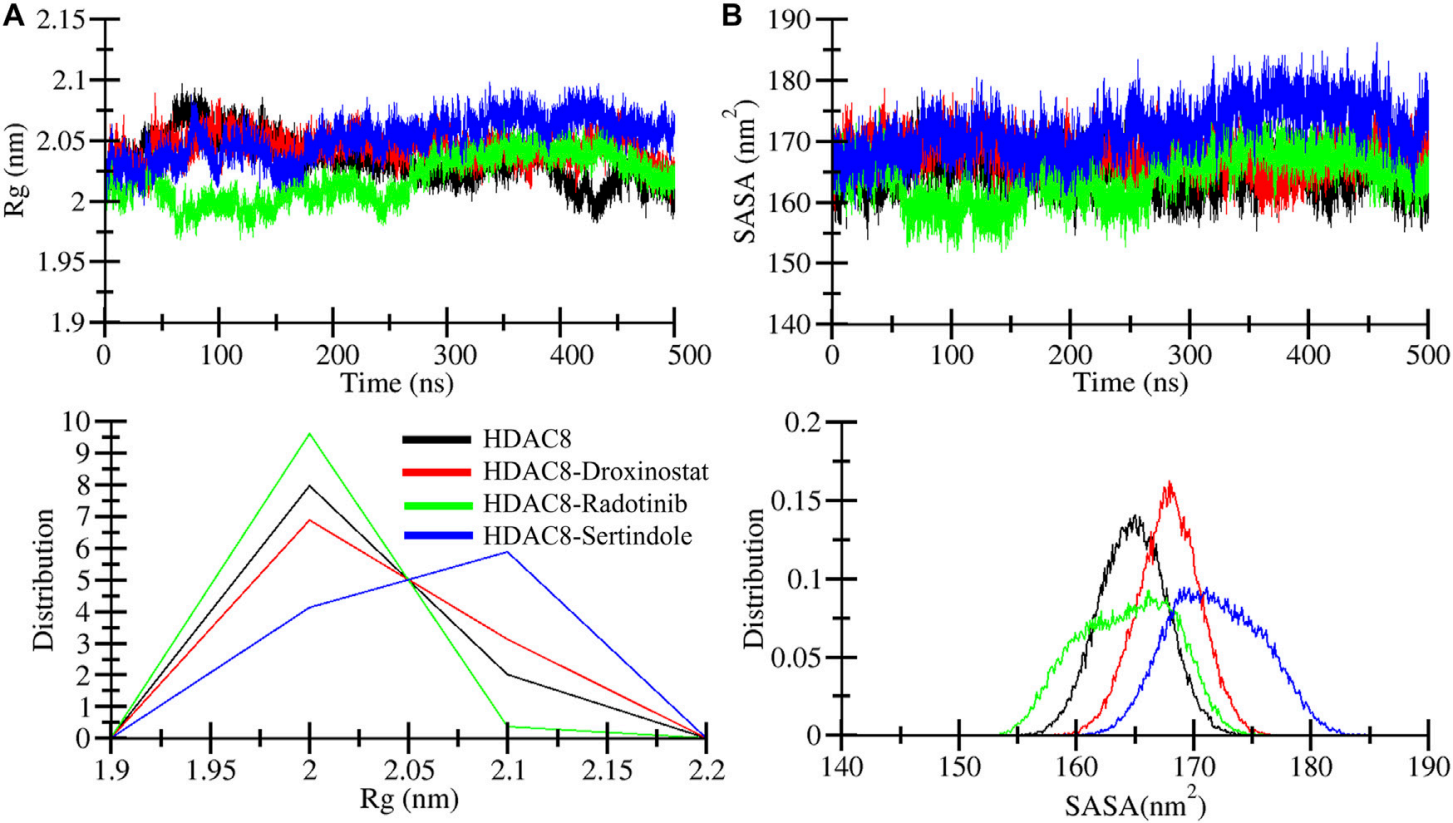
Dynamics of structural compactness. **(A)** Structural compactness derivation by *R*g curves of HDAC8 and HDAC8–ligand docked complex. **(B)** Surface area calculation as SASA of HDAC8 and HDAC8–ligand docked complex. The lower panels show the distributed RMSD and RMSF profiles.

The radius of gyration (*R*g) is used to explore the structural compactness profile of proteins (Lobanov et al., 2008). Higher *R*g values are associated with more unstable or unfolded structures, and the lower *R*g directly belongs to the strong compactness and rigidity of the structure (Hong and Lei, 2009). The MD simulation provides insights to measure the effects of ligand bindings upon protein conformations. In Figure 4A, we illustrated drug molecule interaction effects on the HDAC8 protein, and calculated average gyration values of HDAC8, HDAC8–droxinostat, HDAC8–radotinib, and HDAC8–sertindole systems were 2.03 nm, 2.04 nm, 2.01 nm, and 2.05 nm, respectively (Table 3). The maximum *R*g value point was touched by the HDAC8–sertindole complex, which is like free HDAC8 protein. Except for a minor deviation in the plot of the HDAC8–sertindole complex, which was between 350 ns and 450 ns shown in blue, all complex plots were in decreased and similar patterns. The PDF plot in Figure 4A, lower panel, illustrates a similar distribution of *R*g values except for little variation in the HDAC8–sertindole complex. The resulting trajectory analysis exhibits an association with stability.

The solvent-accessible surface area (SASA) tends to evaluate the binding effect upon the surface area of protein that interacts with a solvent which might decrease or increase during simulation (Ausaf Ali et al., 2014). The surface was measured by analyzing the 500 ns simulation trajectory using the *sasa* module. Figure 4B illustrates a plot showing comparative changes in the surface area of the protein structure. The HDAC8-sertindole complex plot in blue indicates overlapping till 200 ns over HDAC8 protein and HDAC8-droxinostat reference complex and after 200 ns minor increment was seen in the SASA plot. The HDAC8-radotinib complex plot was lower than the free HDAC8 and HDAC8-droxinostat reference complex till 300 ns. Later it got a slight drift still similar protein and reference complex. The average SASA values of HDAC8, HDAC8-droxinostat, HDAC8-radotinib, and HDAC8-sertindole systems were 164.8 nm^2^, 167.8 nm^2^, 164.2 nm^2^, and 171.8 nm^2^, respectively (Table 3). The distribution plot in Figure 4B, lower panel, averaging SASA values indicates no major effect on HDAC8 after ligand binding.

### Hydrogen bond dynamics within protein and between protein–ligand complexes

**FIGURE 5:**
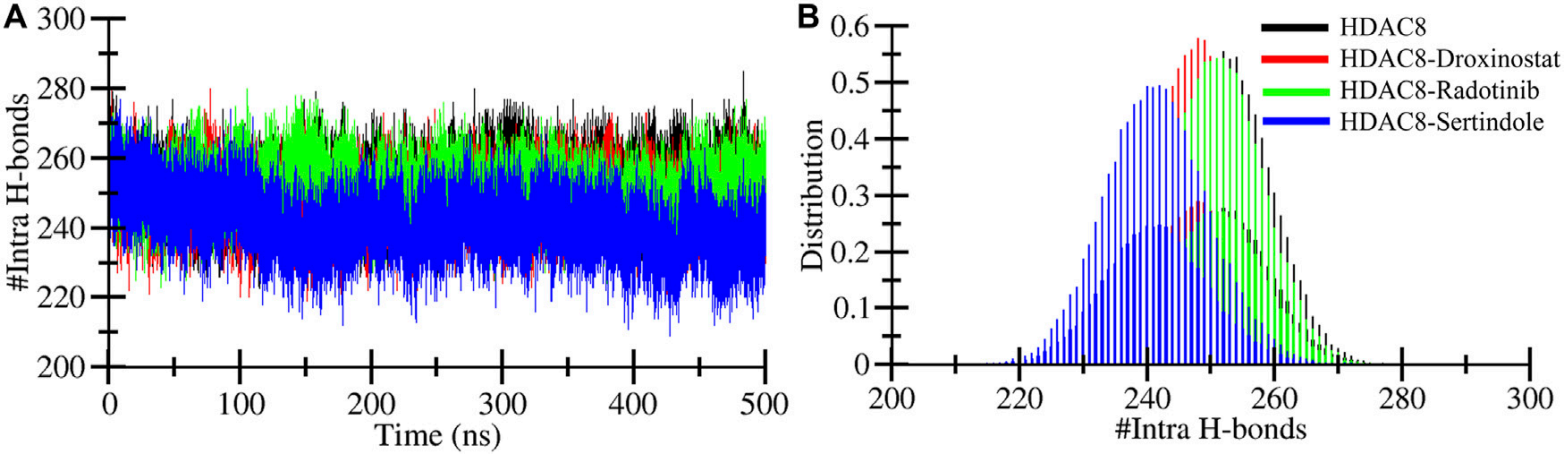
Intramolecular hydrogen bonding. **(A)** Intramolecular hydrogen bond profiles during 500 MD simulation within HDAC8 before and after ligand interactions. **(B)** PDF plot of intramolecular hydrogen bond profiles.

The formation and breaking of hydrogen bonds within the protein are very crucial as they provide structural stability, conformational shape, and three-dimensional functionality (Hubbard and Haider, 2010). Here, we computed hydrogen bonds in the bound and unbound states of the HDAC8 protein. The unbound HDAC8 protein formed 252 average bonds, whereas when bound with droxinostat, radotinib, and sertindole, the total number of bonds was calculated to be 247, 251, and 241, respectively (Table 3). Figure 5A illustrates the bond order of all the systems during the simulation. The HDAC8–radotinib complex plot in green was seen to almost overlap throughout the simulation with the HDAC8 unbound protein and the HDAC8–droxinostat reference complex. The blue plot of the HDAC8–sertindole complex was observed in little down order due to interaction. PDF plot in Figure 5B profiling certain points of hydrogen bonds with their distributing range is observed.

**FIGURE 6:**
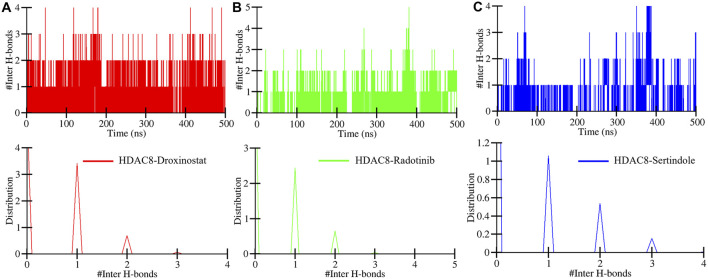
Number of intermolecular hydrogen bonds formed between **(A)** HDAC8–droxinostat, **(B)** HDAC8–radotinib, and **(C)** HDAC8–sertindole.

Intermolecular hydrogen bonds between HDAC8–droxinostat, HDAC8–radotinib, and HDAC8–sertindole complexes were also computed to ensure their stability during simulation. Maximum hydrogen bond formation between HDAC8–droxinostat, HDAC8–radotinib, and HDAC8–sertindole complexes were 4, 5, and 4, respectively. Figure 6 illustrates the number of hydrogen bonds with their time duration. The HDAC8–droxinostat forms 1–4 intermolecular hydrogen bonds where 1–2 bonds show considerably high stability (Figure 6A). At the same time, the HDAC8–radotinib complex forms 1–5 intermolecular hydrogen bonds where 1–2 bonds show considerably high stability (Figure 6B). Similarly, the HDAC8–sertindole complex also forms 1–4 intermolecular hydrogen bonds where 1–2 bonds show considerably high stability (Figure 6C). One hydrogen bond was kept for a long duration in between all three protein–compound complexes, which is also clearly indicated by the distribution plot in Figure 6, lower panels.

### Secondary structure changes examination

**FIGURE 7:**
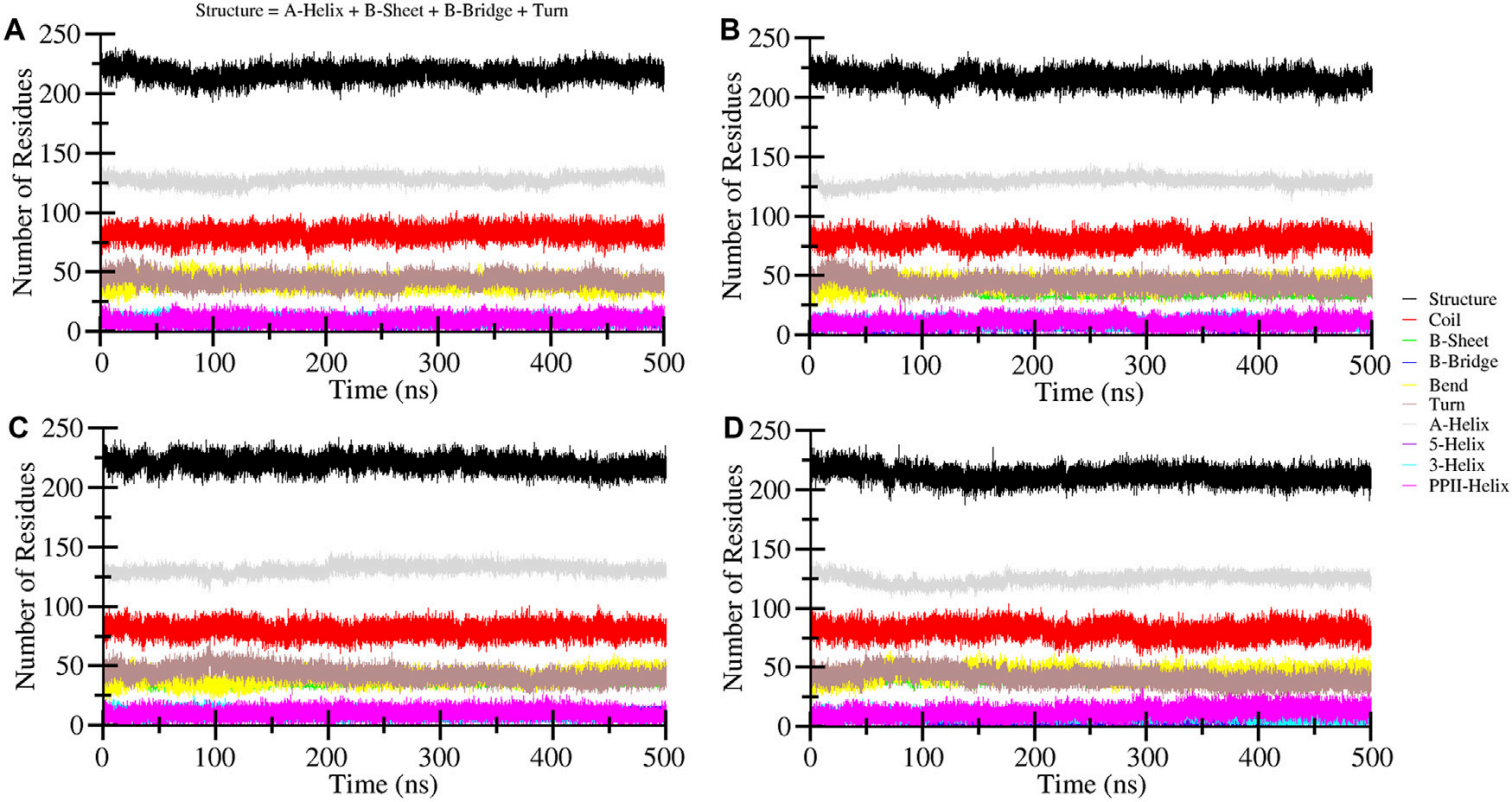
Time-evolution dynamics of the secondary structure during 500 ns MD simulation of **(A)** HDAC8, **(B)** HDAC8–droxinostat, **(C)** HDAC8–radotinib, **(D)** and HDAC8–sertindole.

Changes in the secondary structure content of the HDAC8 protein were computed to study the structural behavior of HDAC8 in the ligand-bound states. For the secondary structure analysis, the DSSP program was utilized with gmx module. The secondary structure content of HDAC8 in unbound and bound states with droxinostat, radotinib, and sertindole is illustrated in Figure 7. Different colors correspond to elements of the HDAC8 protein, and the result showed that radotinib and sertindole molecules did not affect significantly. A little fluctuation in coil and β-sheet formation was observed in the radotinib bound state which is similar to the droxinostat reference molecule bound state (Table 4). Minor residual reduction in structure formation was seen in the sertindole bound state. In the comparison of HDAC8 protein co-crystal reference molecule droxinostat bound state, no major worsened effect was observed after binding of radotinib and sertindole throughout the simulation (Figures 7A–D). This evaluation emphasizes the stability of the protein structure upon drug binding.

**TABLE 4:** Changes that occurred in secondary structure elements during MD simulation were computed.

System	Structure	Coil	β-sheet	β-bridge	Bend	Turn	α-helix	Pi-helix	3_10_-helix	PPII-helix
HDAC8	0.60	0.23	0.11	0.02	0.11	0.12	0.35	0.00	0.03	0.03
HDAC8–droxinostat	0.60	0.22	0.10	0.02	0.12	0.12	0.36	0.00	0.03	0.03
HDAC8–radotinib	0.61	0.22	0.10	0.03	0.11	0.12	0.36	0.00	0.03	0.03
HDAC8–sertindole	0.59	0.23	0.11	0.02	0.12	0.12	0.35	0.00	0.03	0.04

### Principal component analysis

**FIGURE 8:**
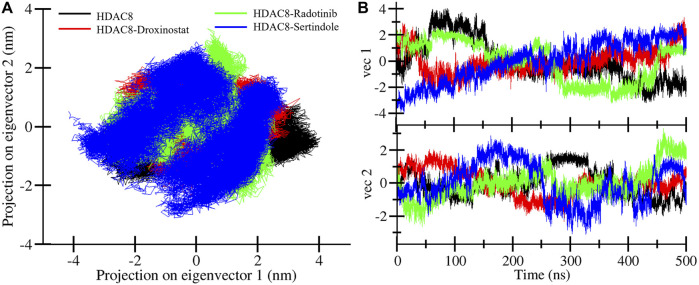
Principal component analysis. **(A)** Projections of trajectories on eigenvectors of HDAC8 bound with droxinostat, radotinib, and sertindole. **(B)** Projection of eigenvector deviation during simulation time.

Principal component analysis is a well-established and proven technique that is used to investigate structural motions during binding (Papaleo et al., 2009). In this study, we have utilized PCA to analyze the motions of HDAC8 protein before and after the binding of drug molecules (Figure 8). The superimposed PCA plots of HDAC8, HDAC8–droxinostat, HDAC8–radotinib, and HDAC8–sertindole complexes are depicted in Figure 8A. Here, all complexes share similar patterns of motions and almost overlap with unbound HDAC8 plots in black. The covered subspace of HDAC8 protein at PC1 and PC2 was −3.1 nm to 4.06 nm and −1.9 nm to 2 nm, respectively. The HDAC8–droxinostat complex covered space at PC1 and PC2 was −2.6 nm to 3.3 nm and −2.3 nm to 2.03 nm, respectively. For the HDAC8–radotinib complex, the calculated subspace at PC1 and PC2 was −3.2 nm to 2.6 nm and −2.6 nm to 3.3 nm, respectively. The HDAC8–sertindole complex covered the area of motion at PC1 and PC2 was −4.1 nm to 2.9 nm and −3.0 nm to 2.8 nm, respectively. Calculated findings showed no major difference was seen even after the binding of drug molecules. Moreover, eigenvector projections with time evolution were also plotted. Figure 8B shows minor fluctuations of HDAC8–radotinib and HDAC8–sertindole complexes over HDAC8 protein and the HDAC8–droxinostat reference complex.

### Free energy landscape analysis

**FIGURE 9:**
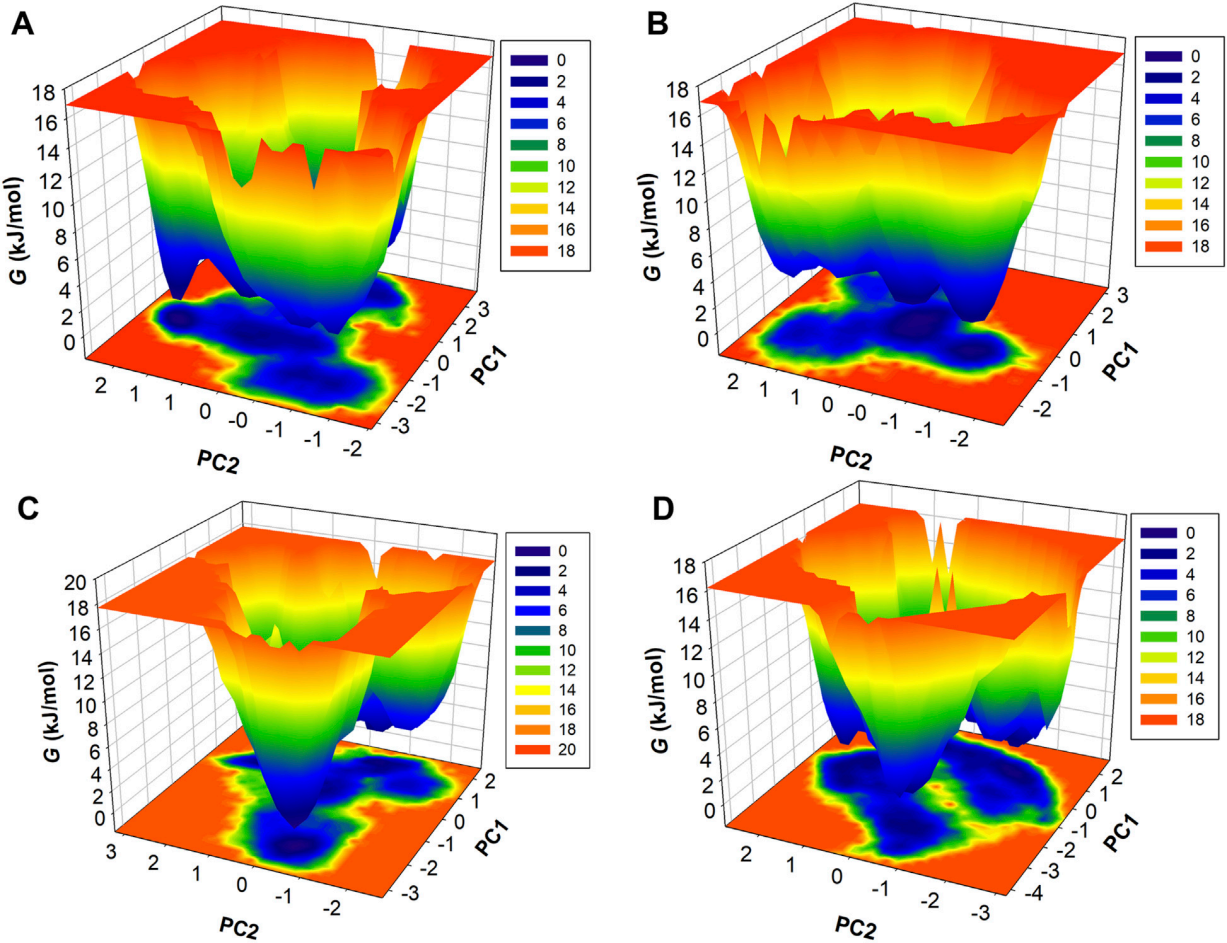
Contour maps of FEL profiles of **(A)** HDAC8, **(B)** HDAC8–droxinostat, **(C)** HDAC8–radotinib, and **(D)** HDAC8–sertindole.

FELs were also generated through PCA components of protein and complexes concerning energy variation. Figure 9 illustrates three-dimensional Gibbs free energy maps where the wider red area is associated with high energy and dark blue belongs to the lowest energy of the systems. The energy range of the HDAC8 protein and the HDAC8–droxinostat reference complex was similar, 0–16.9 kJ/mol, and HDAC8–radotinib and HDAC8–sertindole complexes range 0–17.7 kJ/mol and 0–16.2 kJ/mol, respectively. The HDAC8–sertindole complex possesses wider proportions of a dark blue shade which is associated with favorable conformations. The HDAC8–radotinib complex map also shows favorable conformation associated with a dark blue shade. The HDAC8 and HDAC8–droxinostat complex maps in Figures 9A, B have indicated multiple dark blue basins. The HDAC8–radotinib complex has 3–4 basins which indicate conformational meta states (Figure 9C). Sharp peaks of the HDAC8–sertindole complex in Figure 9D show more stability. Overall, comparative energy map investigation strongly recommended that both HDAC8–drug complexes were stable. Overall, the study indicates that radotinib and sertindole have promising binding potential with stability with HDAC8 and have appropriate drug profiles to be exploited as repurposed drugs in therapeutic development in cancer and neuropathological conditions.

### Radotinib and sertindole potential as HDAC8 repurposed inhibitors and their limitations

Although this study demonstrates the high potential of radotinib and sertindole as HDAC8 inhibitors based on *in silico* approaches, we acknowledge the lack of *in vitro* patient-derived data. Several HDAC8 inhibitors have shown promise in cancer and neurodegenerative diseases in preclinical models (Chakrabarti et al., 2016). The presented results can serve as a basis for further development of repurposed drugs against cancer and neurodegenerative diseases. In future research, patient-derived cell lines should be used to confirm our findings in a more biologically relevant model. Although *in vivo* experiments were not carried out in this study, the action of radotinib and sertindole as HDAC8 inhibitors is in congruity with other small-molecule inhibitors that have been effective in murine cancer models (Ahn, 2018; Lu et al., 2007; Rettig et al., 2015). These results thus support the plausibility we have proposed of both radotinib and sertindole being active *in vivo*, which is important future development research.

In the present study, we demonstrate that radotinib and sertindole are effectively bound with HDAC8, which may alter several cellular signaling pathways. HDAC8 is involved in the regulation of histone acetylation, and consequently, gene expression, cell cycle, and apoptosis. This modulation is important in controlling tumor formation and spread as HDAC8 is involved in cancer development. Moreover, HDAC8 inhibition may also prevent neurotoxicity by regulating histone acetylation in neurons and preventing neurodegenerative diseases (Geng et al., 2023; Fontana et al., 2022). The identification of the specific molecular targets of these inhibitors and the mapping of their signaling networks remain an important area for further investigation. Despite the promising results, several limitations exist. First, the absence of direct experimental validation, including *in vitro* patient-derived data and *in vivo* studies, restricts the translational impact of these findings. Future research should focus on conducting such studies to further confirm the clinical applicability of radotinib and sertindole. Moreover, investigating their pharmacokinetic and pharmacodynamic profiles will provide insight into their therapeutic potential and safety.

## Conclusion

HDAC8 is a class I histone deacetylase that targets chromatin structure and gene expression for modulating disease development and progression. HDAC8 is overexpressed and dysregulated in several types of cancer and neurodegenerative disorders, which calls for potent and selective inhibitors with minimal side effects. By using an integrated virtual screening technique with the help of the DrugBank database, we shortlisted two potential drug molecules, namely, radotinib and sertindole, which exhibited inhibitory effects on HDAC8. These molecules came out as the best hits based on their binding energies, biological activities, and interaction studies. Both drugs showed better binding to HDAC8 than reference inhibitor droxinostat and had considerable interactions with the key residues of the binding site of HDAC8. MD simulation also confirmed the stability and effectiveness of these drug–protein complexes. The analyses of the complexes showed that the complexes of HDAC8 with radotinib and sertindole were quite stable up to 500 ns with good binding interactions and negligible structural fluctuations. The calculations of the essential dynamics also supported these results and confirmed that both complexes stayed stable with minimal dynamics during the time of the simulation. In conclusion, radotinib and sertindole offer potential direction for further research as HDAC8 inhibitors. Further research should be conducted to confirm these results by experimental investigations.

## Data Availability

The original contributions presented in the study are included in the article/Supplementary Material; further inquiries can be directed to the corresponding author.
